# Identification of Drug Interaction Adverse Events in Patients With COVID-19: A Systematic Review

**DOI:** 10.1001/jamanetworkopen.2022.7970

**Published:** 2022-04-19

**Authors:** Valeria Conti, Carmine Sellitto, Martina Torsiello, Valentina Manzo, Emanuela De Bellis, Berenice Stefanelli, Nicola Bertini, Maria Costantino, Chiara Maci, Emanuel Raschi, Francesco Sabbatino, Graziamaria Corbi, Pasquale Pagliano, Amelia Filippelli

**Affiliations:** 1 Department of Medicine, Surgery, and Dentistry, Scuola Medica Salernitana, University of Salerno, Baronissi, Italy; 2 Clinical Pharmacology Unit, San Giovanni di Dio e Ruggi d’Aragona University Hospital, Salerno, Italy; 3 Doctoral School, Department of Medicine, Surgery and Dentistry “Scuola Medica Salernitana,” University of Salerno, Baronissi, Italy; 4 Postgraduate Department of Medicine, Surgery and Dentistry “Scuola Medica Salernitana,” University of Salerno, Baronissi, Italy; 5 Department of Medical and Surgical Sciences, Alma Mater Studiorum, University of Bologna, Bologna, Italy; 6 Oncology Unit, San Giovanni di Dio e Ruggi d’Aragona University Hospital, Salerno, Italy; 7 Department of Medicine and Health Sciences, University of Molise, Campobasso, Italy; 8 Infectious Diseases Unit, San Giovanni di Dio e Ruggi d’Aragona University Hospital, Salerno, Italy

## Abstract

**Importance:**

During the COVID-19 pandemic, urgent clinical management of patients has mainly included drugs currently administered for other diseases, referred to as repositioned drugs. As a result, some of these drugs have proved to be not only ineffective but also harmful because of adverse events associated with drug-drug interactions (DDIs).

**Objective:**

To identify DDIs that led to adverse clinical outcomes and/or adverse drug reactions in patients with COVID-19 by systematically reviewing the literature and assessing the value of drug interaction checkers in identifying such events.

**Evidence Review:**

After identification of the drugs used during the COVID-19 pandemic, the drug interaction checkers Drugs.com, COVID-19 Drug Interactions, LexiComp, Medscape, and WebMD were consulted to analyze theoretical DDI-associated adverse events in patients with COVID-19 from March 1, 2020, through February 28, 2022. A systematic literature review was performed by searching the databases PubMed, Scopus, and Cochrane for articles published from March 1, 2020, through February 28, 2022, to retrieve articles describing actual adverse events associated with DDIs. The drug interaction checkers were consulted again to evaluate their potential to assess such events.

**Findings:**

The DDIs identified in the reviewed articles involved 46 different drugs. In total, 575 DDIs for 58 drug pairs (305 associated with at least 1 adverse drug reaction) were reported. The drugs most involved in DDIs were lopinavir and ritonavir. Of the 6917 identified studies, 20 met the inclusion criteria. These studies, which enrolled 1297 patients overall, reported 115 DDI-related adverse events: 15 (26%) were identifiable by all tools analyzed, 29 (50%) were identifiable by at least 1 of them, and 14 (24%) remained nonidentifiable.

**Conclusions and Relevance:**

The main finding of this systematic review is that the use of drug interaction checkers could have identified several DDI-associated adverse drug reactions, including severe and life-threatening events. Both the interactions between the drugs used to treat COVID-19 and between the COVID-19 drugs and those already used by the patients should be evaluated.

## Key Points

**Question:**

Is it possible to assess adverse events associated with drug-drug interactions (DDIs) by drug interaction checkers in patients with COVID-19?

**Findings:**

The DDIs identified in this systematic review involved 46 different drugs, with 575 DDIs for 58 drug pairs (305 associated with at least 1 adverse drug reaction) reported. Drug interaction checkers could have identified such events, including severe and life-threatening ones.

**Meaning:**

Notwithstanding the emergency context of the COVID-19 pandemic, DDI-related adverse events should never be overlooked to customize the most effective and safest therapy.

## Teaser

This systematic review explores drug-drug interactions associated with adverse clinical outcomes and/or adverse drug reactions in patients with COVID-19 and assesses whether drug interaction checkers are helpful in identifying such events.

## Introduction

The COVID-19 pandemic has overwhelmed a completely unprepared world. Physicians have been faced with the challenge of caring for infected patients in the absence of consolidated scientific evidence and guidelines.^1^ As a consequence, they have used drugs already approved for other diseases, referred to as repositioned drugs.^1,2^ Especially at the beginning of the pandemic, the potential efficacy of these repositioned drugs against SARS-CoV-2 was often based on in vitro or in vivo evidence.^3^ Some of these drugs have been used without considering their potential to cause adverse outcomes associated with drug-drug interactions (DDIs).^4,5^

Drug-drug interactions, determined by pharmacokinetic and pharmacodynamic mechanisms, occur with high frequency in polytreated patients, such as patients with COVID-19.^5^ The increase in adverse outcomes associated with DDIs and/or adverse drug reactions (ADRs) leads to increased hospital admissions and health care costs. Therefore, it is essential to avoid potential DDIs when establishing therapy. Drug interaction checkers are tools used to identify potential DDIs, supporting safe prescribing. This study aimed to identify DDIs that led to adverse clinical outcomes and/or ADRs in patients with COVID-19 by systematically reviewing the literature and assessing the value of drug interaction checkers in identifying such events.

## Methods

The study design for this systematic review involved 4 steps. Step 1 involved the identification of all drugs used during the pandemic by consulting the European Medicines Agency and the Italian Medicines Agency websites, ClinicalTrials.gov database, and literature data. Step 2 involved searching for potential DDIs that involved each drug identified in step 1 using the following drug interaction checkers: Drugs.com, COVID-19 Drug Interactions, LexiComp, Medscape, and WebMD. Step 3 involved a literature systematic review to identify articles that reported adverse clinical outcomes and/or ADRs related to DDIs among COVID-19 treatments and with coadministered drugs. Step 4 involved evaluating whether the DDIs identified in step 3 could have been identified by using the tools listed in step 2.

### Systematic Review

To conduct a comprehensive systematic literature search, we used both controlled vocabulary and free-text terms. The following Medical Subject Heading terms were applied by using the Boolean operator AND: DDIs, COVID-19, patients with COVID-19, comedications, and ADRs. The PubMed, Scopus, and Cochrane databases were searched from the pandemic inception (March 1, 2020) up to February 28, 2022. Our research was limited to articles that involved patients with COVID-19 without sex and age restriction. Articles of any language that identified potential associations between DDIs and relevant clinical outcomes in patients with COVID-19 were included. A systematic review was performed, which identified 6917 studies, following the recommendations of the Meta-analysis of Observational Studies in Epidemiology (MOOSE) reporting guideline^6^ and the PRISMA statement of reporting systematic review and meta-analysis.^7^ This study did not need approval from an ethics committee or written informed consent from patients because it is a systematic review without meta-analysis.

Inclusion criteria were as follows: articles involving patients with a diagnosis of COVID-19, case reports and case series, letters to the editor and communications, observational studies, and interventional clinical trials. Exclusion criteria were as follows: articles that did not report a direct association between DDIs and clinically relevant outcomes in patients with COVID-19, reviews and meta-analyses, conference papers and book chapters, and studies in silico or based on in vitro experiments.

### Drug Interaction Checkers

The drug interaction checkers used in this study were Drugs.com, COVID-19 Drug Interactions, LexiComp, Medscape, and WebMD. Drugs.com^8^ generates a list of DDIs that are marked by a colored dot. Major DDIs (highly clinically significant; avoid combinations) are in red, moderate DDIs (moderately clinically significant; usually avoid combinations; use it only under special circumstances) are in orange, and minor DDIs (minimally clinically significant; minimize risk; assess risk and consider an alternative drug; take steps to circumvent the interaction risk and/or institute a monitoring plan) are in yellow. In COVID-19 Drug Interactions (made by Liverpool University),^9^ the drugs are divided according to the risk of clinically significant interaction as follows: do not coadminister (with a red circle), potential interaction (with an orange square), potential weak interaction (with a yellow triangle), and no interaction expected (with a green rhombus). The LexiComp interactions tool^10^ identifies DDIs, assigning the following risk rating: A, no known interaction; B, no action needed; C, monitor therapy; D, consider therapy modification; and X, avoid combination. LexiComp reports the drug class and the mechanism responsible for the interaction. The Medscape tool^11^ classifies the DDIs as follows: contraindicated (in red), serious–use alternative (in orange), monitor closely (in green), and minor (in blue). The degree of severity is indicated by different shades of red: contraindicated (in dark red), serious–use alternative (in red), monitor closely (in pink), and minor (in light pink). The WebMD tool^12^ classifies the DDI risk as follows: don’t use together (in red), serious (in orange), monitor closely (in yellow), and minor (in green).

## Results

### Identification of Drugs Used During the COVID-19 Pandemic and Potential DDIs

The drugs used during the COVID-19 pandemic were identified by consulting the website of the European Medicines Agency,^13^ Italian Medicines Agency,^14^ ClinicalTrials.gov,^15^ and literature data (Figure). The Figure shows all the 46 drugs listed in chronological order according to their period of use. Most of them were used under the concept of repurposing; some have been included in clinical trials or administered as off-label or compassionate use.

**Figure.**
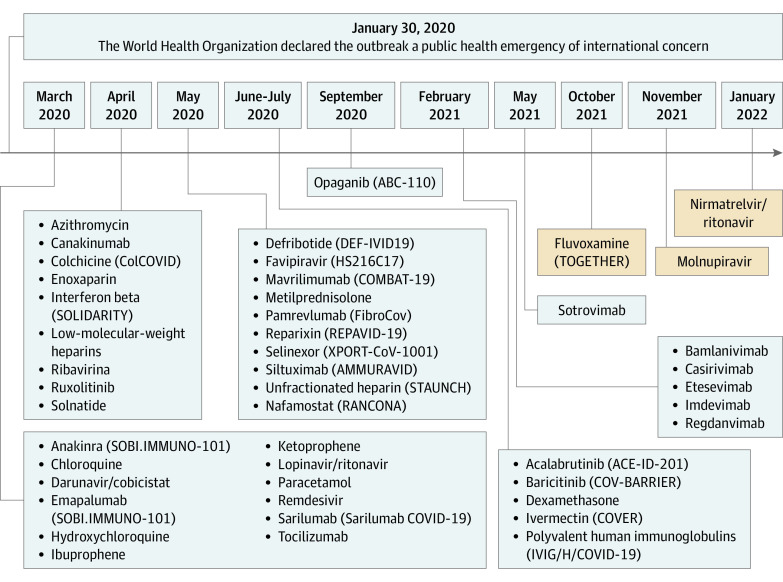
Timeline of the Drugs Used During the COVID-19 Pandemic ABC-110 indicates Study of Opaganib in Coronavirus Disease 2019 Pneumonia (COVID-19); ACE-ID-201, A Phase 2, Open Label, Randomized Study of the Efficacy and Safety of Acalabrutinib with Best Supportive Care Vs Best Supportive Care in Subjects Hospitalized with COVID-19; AMMURAVID, Factorial Randomized Trial of Remdesivir and Baricitinib Plus Dexamethasone for COVID-19; ColCOVID, Colchicine Counteracting Inflammation in COVID-19 Pneumonia; COMBAT-19, Mavrilimumab in Severe COVID-19 Pneumonia and Hyper-inflammation; COV-BARRIER, Study of Baricitinib (LY3009104) in Children With COVID-19; COVER, COVID Ivermectin - Randomized, Double-blind, Multi Centre Phase II, Proof of Concept, Dose Finding Clinical Trial on Ivermectin for the Early Treatment of COVID-19; DEF-IVID19, Defibrotide in COVID-19 Pneumonia - Use of Defibrotide to Reduce Progression of Acute Respiratory Failure Rate in Patients With COVID-19 Pneumonia; FibroCov, Open-label, Randomized, Parallel-arm Study Investigating the Efficacy and Safety of Intravenous Administration of Pamrevlumab Vs Standard of Care in Patients With COVID-19; HS216C17, Clinical Study to Evaluate the Performance and Safety of Favipiravir in COVID-19; IVIG/H/COVID-19, High Dose Intravenous Polyvalent Immunoglobulin (IVIG) in Patients With Early Inflammatory COVID-19; RANCONA, A Randomized Clinical Trial of Nafamostat: A Potent Transmembrane Protease Serine 2 (TMPRSS2) Inhibitor for the Treatment of Covid-19; REPAVID-19, Reparixin in COVID-19 Pneumonia - Efficacy and Safety; SOBI.IMMUNO-101, Efficacy and Safety of Emapalumab and Anakinra in Reducing Hyperinflammation and Respiratory Distress in Patients With COVID-19 Infection; SOLIDARITY, Efficacy of Different Anti-viral Drugs in COVID 19 Infected Patients; STAUNCH, Steroids and Unfractionated Heparin in Critically Ill Patients With Pneumonia From COVID-19 Infection; TOGETHER, Trial to Evaluate the Effect of Peginterferon Lambda for the Treatment of COVID-19; XPORT-CoV-1001, Evaluation of Activity and Safety of Oral Selinexor in Participants With Severe COVID-19 Infection.

eTable 1 in the Supplement reports the number of potential DDIs for each drug administered against COVID-19 with the degree of severity of the associated adverse outcomes and/or ADRs found by using the drug interaction checkers. The tools differed from each other regarding the number of potential DDIs identified and the classification of the severity grade of the DDI-associated clinical outcomes. Drugs.com identified the largest numbmer of DDIs, followed by Medscape, WebMD, LexiComp, and COVID-19 Drug Interactions. Drugs.com identified the lirgest number of highly clinically significant DDI-associated adverse outcomes, followed by LexiComp, COVID-19 Drug Interactions, WebMD, and Medscape (eTable 1 in the Supplement).

The drugs most involved in DDIs were lopinavir and ritonavir, followed by nirmatrelvir and ritonavir, darunavir and cobicistat, chloroquine, acetazolamide, and hydroxychloroquine. The drug interaction checkers agree with each other (even if with different classification methods) in considering lopinavir and ritonavir as the drug involved in the most serious DDI-associated ADRs (eTable 1 in the Supplement).

### Systematic Review Results

A systematic review was performed to identify adverse clinical outcomes and/or ADRs related to DDIs among treatments of COVID-19 and between COVID-19 treatments and drugs coadministered in patients with COVID-19. The PRISMA algorithm^7^ shows the research workflow (eFigure in the Supplement). Twenty articles^16,17,18,19,20,21,22,23,24,25,26,27,28,29,30,31,32,33,34,35^ that involved 46 interacting drugs that led to DDI-associated adverse outcomes were evaluated (Table 1). The most frequent DDIs were hydroxychloroquine and lopinavir-ritonavir. Most DDIs resulted in QT-interval prolongation. Such a dangerous alteration was found 20 times, and in 12 of these 20 cases, it occurred together with other adverse outcomes, even leading to the death of 8 patients (Table 1).

**Table 1.** Study Design, DDIs, DDI-Associated Adverse Outcomes, and Mechanisms Reported by the Reviewed Studies

Source	Study design	Study quality score	Drugs involved in potential DDIs	DDIs mechanism	DDI-associated adverse clinical outcomes and/or ADRs
Anmella et al,^16^ 2020	Case series	4	Acetazolamide, hydroxychloroquine, lopinavir-ritonavir, paroxetine, risperidone, and topiramate	PK	Behavioral disturbances
	Case series	4	Acetazolamide, hydroxychloroquine, lopinavir and ritonavir, venlafaxine	PK	Mild QT-interval prolongation (443 ms)
Bartiromo et al,^17^ 2020	Case report	5	Darunavir-cobicistat, hydroxychloroquine, and tacrolimus	PK	Tacrolimus trough levels found to be extremely high (90.5 ng/mL), intermittent abdominal pain, nausea and vomiting
Borba et al,^18^ 2020	Original investigation	1	Acetazolamide, ceftriaxone, and chloroquine	PK	Rhabdomyolysis, myocarditis, severe arrhythmias, QT-interval prolongation
	Original investigation	1	Acetazolamide, ceftriaxone, chloroquine, and oseltamivir	PK	Rhabdomyolysis, myocarditis, severe arrhythmias, QT-interval prolongation
Crescioli et al,^19^ 2021	Observational study	3	Amiodarone, acetazolamide, darunavir-cobicistat, and hydroxychloroquine	PK and PD	QT-interval prolongation
	Observational study	3	Acetazolamide, citalopram, hydroxychloroquine, and lopinavir and ritonavir	PK and PD	QT-interval prolongation
	Observational study	3	Acetazolamide, darunavir-cobicistat, and hydroxychloroquine	PK and PD	Diarrhea, vomiting
	Observational study	3	Acetazolamide, haloperidol, hydroxychloroquine, levomepromazine, lopinavir-ritonavir, and zuclopenthixol	PK and PD	QT-interval prolongation
	Observational study	3	Acetazolamide, haloperidol, hydroxychloroquine, and lopinavir-ritonavir	PK and PD	QT-interval prolongation
	Observational study	3	Acetazolamide, hydroxychloroquine, and lopinavir-ritonavir	PK and PD	QT-interval prolongation
	Observational study	3	Acetazolamide, hydroxychloroquine, and lopinavir-ritonavir	PK and PD	QT-interval prolongation, vomiting
	Observational study	3	Acetazolamide, hydroxychloroquine, and sertraline	PK and PD	QT-interval prolongation
	Observational study	3	Citalopram and hydroxychloroquine	PK and PD	QT-interval prolongation
	Observational study	3	Darunavir-cobicistat, haloperidol, and hydroxychloroquine	PK and PD	QT-interval prolongation
	Observational study	3	Darunavir-cobicistat and hydroxychloroquine	PK and PD	QT-interval prolongation
	Observational study	3	Darunavir-cobicistat, hydroxychloroquine, lopinavir-ritonavir, and tocilizumab	PK and PD	Hypertransaminasemia
	Observational study	3	Darunavir-cobicistat, hydroxychloroquine, and tocilizumab	PK and PD	Psychosis, agitation, delirium, aggressiveness
	Observational study	3	Darunavir-cobicistat and tacrolimus	PK and PD	Nausea, vomiting, abdominal pain, drug level modification
	Observational study	3	Hydroxychloroquine and lopinavir-ritonavir	PK and PD	QT-interval prolongation, hypokalemia
	Observational study	3	Hydroxychloroquine and lopinavir-ritonavir	PK and PD	QT-interval prolongation
	Observational study	3	Hydroxychloroquine and magnesium sulfate	PK and PD	QT-interval prolongation, hypokalemia
	Observational study	3	Hydroxychloroquine and risperidone	PK and PD	QT-interval prolongation, atrial flutter, hemiplegia, hypokalemia, major depression
	Observational study	3	Hydroxychloroquine and trazodone	PK and PD	QT-interval prolongation
Dajti et al,^20^ 2020	Case report	5	DRV/c, hydroxychloroquine, prednisone, and tacrolimus	PK	Increased tacrolimus levels
Gautret et al,^21^ 2021	Letter	5	Acetazolamide and hydroxychloroquine	PK	QT-interval prolongation (>60 ms), discontinuation of treatment
Ghani et al,^22^ 2020	Case series	4	Apixaban, enoxaparin, hydroxychloroquine, and corticosteroids	PK	A large intraparenchymal hemorrhage and cerebral edema
	Case series	4	Apixaban, hydroxychloroquine, corticosteroids, and unfractionated heparin	PK	Scattered subarachnoid hemorrhages, a subdural hematoma
	Case series	4	Hydroxychloroquine, corticosteroids, and unfractionated heparin	PK	Acute subarachnoid and intraparenchymal hemorrhages within the posterior fossa
Li et al,^23^ 2020	Observational study	3	Ganciclovir, lopinavir-ritonavir, oseltamivir, peramivir, penciclovir, rubavirin, and umifenovir	PK	Increase in D-dimer, hematologic abnormalities
Macías et al,^24^ 2020	Cross-sectional study	4	Amiodarone and lopinavir-ritonavir	PK	Orthostatic syncope
Martínez-López-de-Castro et al,^25^ 2021	Cohort, retrospective and single-center study	3	Alprazolam and lopinavir-ritonavir	PK	Psychiatric disorders
	Cohort, retrospective and single-center study	3	Aripiprazole, digoxin, fentanyl, lithium, lopinavir-ritonavir, and tacrolimus	PK	Alteration of the concentration of blood levels
	Cohort, retrospective and single-center study	3	Acetazolamide and hydroxychloroquine	PK	Cutaneous reactions
	Cohort, retrospective and single-center study	3	Acetazolamide and lopinavir-ritonavir	PK	Gastrointestinal disorders
	Cohort, retrospective and single-center study	3	Hydroxychloroquine and lopinavir-ritonavir	PK	Hyperglycemia
	Cohort, retrospective and single-center study	3	Hydroxychloroquine and lopinavir-ritonavir	PK	Cutaneous reactions
	Cohort, retrospective and single-center study	3	Hydroxychloroquine and lopinavir-ritonavir	PK	Gastrointestinal disorders
	Cohort, retrospective and single-center study	3	Hydroxychloroquine and lopinavir-ritonavir	PK	Psychiatric disorders
	Cohort, retrospective and single-center study	3	Hydroxychloroquine and tacrolimus	PK	Alteration of the concentration of blood levels
	Cohort, retrospective and single-center study	3	Interferon beta and metamizole	PK	Hematologic toxicity
	Cohort, retrospective and single-center study	3	Lopinavir-ritonavir and methylprednisolone or prednisone	PK	Hyperglycemia
	Cohort, retrospective and single-center study	3	Lopinavir-ritonavir and midazolam or diazepam	PK	Increased sedative effect
	Cohort, retrospective and single-center study	3	Lopinavir-ritonavir and propofol	PK	Increased triglyceride level
	Cohort, retrospective and single-center study	3	Lopinavir-ritonavir and simvastatin	PK	Liver toxicity
	Cohort, retrospective and single-center study	3	Lopinavir-ritonavir and valproate	PK	Seizures
Meriglier et al,^26^ 2021	Observational study	2	Darunavir-ritonavir and hydroxychloroquine	PK	Diarrhea grade I and II; ECG abnormalities; hepatic enzyme elevation
	Observational study	2	Hydroxychloroquine and lopinavir-ritonavir	PK	Diarrhea grade I and II; ECG abnormalities; severe nausea
Meziyerh et al,^27^ 2020	Case report	5	Everolimus, hydroxychloroquine, and lopinavir-ritonavir	PK	Dyspnea or tachypnea, everolimus plasma concentrations increased
Nham et al,^28^ 2020	Case report	5	Ceftriaxone, levofloxacin, and lopinavir-ritonavir	PK	Severe thrombocytopenia with epistaxis and petechiae
Ramireddy et al,^29^ 2020	Original research	3	Acetazolamide and hydroxychloroquine	PK	QT-interval prolongation
Skroza et al,^30^ 2020	Case report	5	Ceftriaxone, enoxaparin, hydroxychloroquine, and lopinavir-ritonavir	PK	Urticarial vasculitis attributable to adverse drug reaction
Szekely et al,^31^ 2020	Original research	2	Chloroquine, letrozole, and memantine	PK	Extreme QT-interval prolongation (720 ms), torsades de pointes
Teoli et al,^32^ 2021	Case report	5	Remdesivir and tramadol	PK	Severe pain localized in the legs
Thammathiwat et al,^33^ 2021	Case report	5	Darunavir-ritonavir and tacrolimus	PK	Tacrolimus levels turned significantly high, acute kidney injury, lymphopenia, Pio_2_/Fio_2_ was lowered, tacrolimus withdrawn for 10 d
Treon et al,^34^ 2020	Letter	5	Acetazolamide, hydroxychloroquine, and ibrutinib	PK	Wide QRS complex tachyarrhythmia
Yekedüz et al,^35^ 2020	Case report	5	Antidiabetics and hydroxychloroquine	PK	Hypoglycemia

Abbreviations: ADRs, adverse drug reactions; DDIs, drug-drug interactions; DRV/c, darunavir-cobicistat; ECG, electrocardiography; Fio_2_, fraction of inspired oxygen; PD, pharmacodynamic; Pio_2_, inspired oxygen tension; PK, pharmacokinetic.

Eleven DDI-associated ADRs were diarrhea and vomiting as well as liver disorders. Six neurologic or psychiatric DDIs were reported. Three of 6 were serious neurovascular hemorrhages. One of them involved corticosteroids, hydroxychloroquine, and unfractionated heparin, another one implicated the aforementioned drugs coadministered with apixaban, and the last one included fractionated heparin. Surprisingly, none of the reviewed studies reported DDIs that involved ritonavir and anticoagulants. However, all drug interaction checkers agreed that the most severe DDIs occurred with ritonavir and direct factor Xa inhibitors.

Drugs coadministered with hydroxychloroquine were lopinavir-ritonavir (24 cases), acetazolamide (20 cases), and darunavir-cobicistat (15 cases). Of the 53 DDIs in which hydroxychloroquine was involved, 31 were associated with QT-interval prolongation. Four of these 31 DDIs led to patient death.

Only 3 of 56 DDIs involved coadministration of chloroquine with other drugs, including ceftriaxone, acetazolamide, and oseltamivir^18^ or memantine and letrozole.^31^ The major complication linked to chloroquine and hydroxychloroquine, in monotherapy or in combination and in short or low-dose regimens, was again QT-interval prolongation, which also caused fatal arrhythmias.^18^

### DDI-Associated Clinical Outcomes and/or ADRs Identified by Drug Interaction Checkers

The DDIs identified in the reviewed articles involved 46 different drugs (Table 1). Many of them were administered for patients’ comorbidities. Table 2 lists all the drugs reported in the articles and identified as triggers of DDIs by at least 1 of the drug interaction checkers used. Drugs.com was the most complete tool. Conversely, COVID-19 Drug Interactions, WebMD, Medscape, and LexiComp did not include some medications, such as memantine, letrozole, and magnesium sulfate (Table 2).

**Table 2.** DDIs Reported in the Reviewed Studies and the Results of the 5 Consulted Drug Interaction Checkers

Drugs involved in DDIs reported in the reviewed studies	Drugs.com	COVID-19 Drug Interactions	LexiComp	Medscape	WebMD
Alprazolam and lopinavir-ritonavir	Moderate	Potential interaction	X: Avoid combination	Serious - use alternative	Serious
Amiodarone and DRV/c	Moderate	No interaction found	C: Monitor therapy	Monitor closely	Monitor closely
Amiodarone and hydroxychloroquine	Major	Do not coadminister	No interaction found	Serious - use alternative	Serious
Amiodarone and lopinavir-ritonavir	Major	Do not coadminister	X: Avoid combination	Contraindicated	Don’t use together
Antidiabetics and hydroxychloroquine	Moderate	Potential interaction	C: Monitor therapy	No interaction found	No interaction found
Apixaban and enoxaparin	Major	Potential interaction	X: Avoid combination	Serious - use alternative	Serious
Apixaban and hydroxychloroquine	No interaction found	Potential weak interaction	No interaction found	No interaction found	No interaction found
Apixaban and unfractionated heparin	No interaction found	No interaction found	X: Avoid combination	Serious - use alternative	Serious
Aripiprazole and digoxin	Moderate	No interaction found	No interaction found	No interaction found	No interaction found
Aripiprazole and fentanyl	No interaction found	No interaction found	D: Consider therapy modification	Monitor closely	Monitor closely
Aripiprazole and lithium	No interaction found	No interaction found	C: Monitor therapy	Monitor closely	No interaction found
Aripiprazole and lopinavir-ritonavir	Moderate	Potential interaction	D: Consider therapy modification	Serious - use alternative	Serious
Aripiprazole and tacrolimus	No interaction found	No interaction found	No interaction found	No interaction found	Serious
Acetazolamide and amiodarone	Major	Do not coadminister	D: Consider therapy modification	Serious - use alternative	Monitor closely
Acetazolamide and ceftriaxone	No interaction found	No interaction expected	No interaction found	No interaction found	No interaction found
Acetazolamide and citalopram	Major	Do not coadminister	C: Monitor therapy	Monitor closely	Monitor closely
Acetazolamide and chloroquine	Major	Potential interaction	C: Monitor therapy	Monitor closely	Monitor closely
Acetazolamide and DRV/c	Major	No interaction expected	No interaction found	Serious - use alternative	Serious
Acetazolamide and haloperidol	Major	Do not coadminister	C: Monitor therapy	Monitor closely	Monitor closely
Acetazolamide and hydroxychloroquine	Major	Potential interaction	B: No action needed	Serious - use alternative	Serious
Acetazolamide and levomepromazine	No interaction found	Potential interaction	B: No action needed	No interaction found	No interaction found
Acetazolamide and lopinavir	Moderate	No interaction found	B: No action needed	Monitor closely	No interaction found
Acetazolamide and lopinavir and ritonavir	Moderate	Potential interaction	B: No action needed	Monitor closely	Monitor closely
Acetazolamide and oseltamivir	No interaction found	No interaction expected	No interaction found	No interaction found	No interaction found
Acetazolamide and paroxetine	No interaction found	No interaction expected	No interaction found	Minor	Minor
Acetazolamide and risperidone	No interaction found	No interaction expected	C: Monitor therapy	Minor	No interaction found
Acetazolamide and ritonavir	Moderate	Potential interaction	B: No action needed	Monitor closely	Monitor closely
Acetazolamide and sertraline	Moderate	No interaction expected	C: Monitor therapy	Minor	Minor
Acetazolamide and zenlafaxine	No interaction found	Do not coadminister	No interaction found	Minor	Minor
Acetazolamide and zuclopenthixol	No interaction found	Do not coadminister	B: No action needed	No interaction found	No interaction found
Ceftriaxone and chloroquine	No interaction found	No interaction expected	No interaction found	No interaction found	No interaction found
Ceftriaxone and enoxaparin	Minor	No interaction found	No interaction found	Serious - use alternative	Serious
Ceftriaxone and lopinavir-ritonavir	No interaction found	No interaction expected	No interaction found	No interaction found	No interaction found
Citalopram and hydrochloroquine	Major	Do not coadminister	C: Monitor therapy	Serious - use alternative	Serious
Citalopram and lopinavir-ritonavir	Major	No interaction found	B: No action needed	Monitor closely	Monitor closely
Citalopram and ritonavir	Minor	No interaction found	No interaction found	No interaction found	No interaction found
Cobicistat and ritonavir	Moderate	No interaction found	No interaction found	No interaction found	No interaction found
Chloroquine and oseltamivir	No interaction found	No interaction expected	No interaction found	No interaction found	No interaction found
Darunavir and lopinavir	Moderate	No interaction found	No interaction found	Serious - use alternative	No interaction found
Darunavir and prednisone	Moderate	No interaction found	No interaction found	Monitor closely	Monitor closely
Darunavir and tacrolimus	Major	No interaction found	D: Consider therapy modification	Monitor closely	Monitor closely
Darunavir-ritonavir and hydroxychloroquine	Moderate	Potential weak interaction	No interaction found	No interaction found	No interaction found
Darunavir-ritonavir and tacrolimus	Major	No interaction found	D: Consider therapy modification	Monitor closely	Monitor closely
Darunavir and hydroxychloroquine	Moderate	No interaction found	No interaction found	No interaction found	No interaction found
Darunavir and ritonavir	No interaction found	No interaction found	No interaction found	Serious - use alternative	Serious
Diazepam and lopinavir	No interaction found	No interaction found	No interaction found	Monitor closely	No interaction found
Diazepam and lopinavir and ritonavir	Moderate	Potential interaction	C: Monitor therapy	Monitor closely	Monitor closely
Digoxin and hydroxychloroquine	No interaction found	No interaction found	No interaction found	Serious - use alternative	No interaction found
Digoxin and lopinavir and ritonavir	Moderate	No interaction found	No interaction found	No interaction found	No interaction found
Digoxin and tacrolimus	No interaction found	No interaction found	No interaction found	Monitor closely	Monitor closely
Darunavir-cobicistat and haloperidol	Moderate	No interaction found	No interaction found	Monitor closely	Monitor closely
Darunavir-cobicistat and hydroxychloroquine	Moderate	Potential weak interaction	No interaction found	No interaction found	No interaction found
Darunavir-cobicistat and lopinavir-ritonavir	No interaction found	Do not coadminister	No interaction found	No interaction found	Serious
Darunavir-cobicistat and tacrolimus	Major	No interaction found	D: Consider therapy modification	Monitor closely	Monitor closely
Darunavir-cobicistat and tocilizumab	No interaction found	No interaction expected	No interaction found	No interaction found	No interaction found
Enoxaparin and hydroxychloroquine	No interaction found	No interaction expected	No interaction found	No interaction found	No interaction found
Enoxaparin and lopinavir-ritonavir	No interaction found	No interaction expected	No interaction found	No interaction found	No interaction found
Enoxaparin and corticosteroids	No interaction found	No interaction found	No interaction found	Monitor closely	Monitor closely
Everolimus and hydroxychloroquine	No interaction found	No interaction found	No interaction found	Serious - use alternative	No interaction found
Everolimus and lopinavir-ritonavir	No interaction found	No interaction found	X: Avoid combination	Serious - use alternative	No interaction found
Fentanyl and lopinavir	No interaction found	No interaction found	No interaction found	Serious - use alternative	No interaction found
Fentanyl and lopinavir-ritonavir	Major	Potential interaction	D: Consider therapy modification	Serious - use alternative	No interaction found
Ganciclovir and peramivir	No interaction found	No interaction found	No interaction found	Monitor closely	Monitor closely
Haloperidol and hydroxychloroquine	Major	Do not coadminister	C: Monitor therapy	Serious - use alternative	Serious
Haloperidol and lopinavir	No interaction found	No interaction found	C: Monitor therapy	Serious - use alternative	No interaction found
Haloperidol and lopinavir-ritonavir	Major	Do not coadminister	C: Monitor therapy	Serious - use alternative	Serious
Haloperidol and ritonavir	Moderate	No interaction found	No interaction found	No interaction found	No interaction found
Haloperidol and zuclopenthixol	No interaction found	No interaction found	C: Monitor therapy	No interaction found	No interaction found
Hydroxychloroquine and levomepromazine	No interaction found	Potential interaction	No interaction found	No interaction found	No interaction found
Hydroxychloroquine and lopinavir	Major	Potential interaction	No interaction found	Serious - use alternative	No interaction found
Hydroxychloroquine and lopinavir and ritonavir	Major	Potential interaction	No interaction found	Serious - use alternative	Serious
Hydroxychloroquine and magnesium sulfate	Moderate	No interaction found	No interaction found	No interaction found	No interaction found
Hydroxychloroquine and paroxetine	No interaction found	Potential interaction	C: Monitor therapy	No interaction found	No interaction found
Hydroxychloroquine and prednisone	No interaction found	No interaction expected	No interaction found	No interaction found	No interaction found
Hydroxychloroquine and risperidone	Major	Potential interaction	B: No action needed	Serious - use alternative	Serious
Hydroxychloroquine and ritonavir	Moderate	No interaction found	No interaction found	Serious - use alternative	Serious
Hydroxychloroquine and sertraline	Major	No interaction expected	No interaction found	Serious - use alternative	Serious
Hydroxychloroquine and corticosteroids	No interaction found	No interaction expected	No interaction found	No interaction found	No interaction found
Hydroxychloroquine and tacrolimus	Major	Potential interaction	No interaction found	Serious - use alternative	Serious
Hydroxychloroquine and tocilizumab	Moderate	Potential interaction	No interaction found	Serious - use alternative	Serious
Hydroxychloroquine and topiramate	Moderate	No interaction expected	No interaction found	No interaction found	No interaction found
Hydroxychloroquine and trazodone	Major	Potential interaction	No interaction found	No interaction found	No interaction found
Hydroxychloroquine and unfractionated heparin	No interaction found	No interaction expected	No interaction found	Monitor closely	No interaction found
Hydroxychloroquine and venlafaxine	Major	Do not coadminister	No interaction found	No interaction found	No interaction found
Hydroxychloroquine and zuclopenthixol	No interaction found	Do not coadminister	No interaction found	No interaction found	No interaction found
Interferon beta and metamizole	No interaction found	Do not coadminister	No interaction found	No interaction found	No interaction found
Levofloxacin and lopinavir-ritonavir	No interaction found	Potential interaction	No interaction found	No interaction found	No interaction found
Levomepromazine and lopinavir-ritonavir	No interaction found	Potential interaction	No interaction found	No interaction found	No interaction found
Lithium and lopinavir-ritonavir	Moderate	Potential interaction	No interaction found	No interaction found	No interaction found
Lopinavir and methylprednisolone	No interaction found	No interaction found	No interaction found	Monitor closely	No interaction found
Lopinavir and midazolam	No interaction found	No interaction found	No interaction found	Serious - use alternative	No interaction found
Lopinavir and prednisone	No interaction found	No interaction found	No interaction found	Monitor closely	No interaction found
Lopinavir and tacrolimus	No interaction found	No interaction found	No interaction found	Serious - use alternative	No interaction found
Lopinavir and venlafaxine	No interaction found	No interaction found	No interaction found	Monitor closely	No interaction found
Lopinavir-ritonavir and methylprednisolone	Major	No interaction expected	C: Monitor therapy	Serious - use alternative	Serious
Lopinavir-ritonavir and midazolam	Major	Do not coadminister	X: Avoid combination	Serious - use alternative	Serious
Lopinavir-ritonavir and paroxetine	Moderate	Potential interaction	No interaction found	No interaction found	No interaction found
Lopinavir-ritonavir and prednisone	Moderate	Potential interaction	C: Monitor therapy	Monitor closely	Monitor closely
Lopinavir-ritonavir and propofol	Moderate	Potential interaction	No interaction found	No interaction found	No interaction found
Lopinavir-ritonavir and risperidone	Moderate	Potential interaction	C: Monitor therapy	No interaction found	No interaction found
Lopinavir-ritonavir and simvastatin	Major	Potential interaction	No interaction found	Contraindicated	Don’t use together
Lopinavir-ritonavir and tacrolimus	Major	Potential interaction	D: Consider therapy modification	Serious - use alternative	Serious
Lopinavir-ritonavir and topiramate	No interaction found	No interaction found	No interaction found	Monitor closely	Monitor closely
Lopinavir-ritonavir and valproate	Moderate	Potential interaction	C: Monitor therapy	No interaction found	No interaction found
Lopinavir-ritonavir and venlafaxine	Moderate	Potential interaction	B: No action needed	No interaction found	Monitor closely
Lopinavir-ritonavir and zuclopenthixol	No interaction found	Potential interaction	No interaction found	No interaction found	No interaction found
Methylprednisolone and ritonavir	Major	No interaction found	No interaction found	No interaction found	No interaction found
Paroxetine and risperidone	No interaction found	No interaction found	D: Consider therapy modification	Monitor closely	Monitor closely
Paroxetine and topiramate	No interaction found	No interaction found	B: No action needed	No interaction found	No interaction found
Prednisone and ritonavir	Moderate	No interaction found	No interaction found	Monitor closely	Monitor closely
Prednisone and tacrolimus	Moderate	No interaction found	C: Monitor therapy	Minor	Minor
Remdesivir and tramadol	No interaction found	No interaction expected	C: Monitor therapy	No interaction found	No interaction found
Risperidone and topiramate	No interaction found	No interaction found	C: Monitor therapy	Monitor closely	Monitor closely
Ritonavir and tacrolimus	Major	No interaction found	D: Consider therapy modification	Monitor closely	Monitor closely
Corticosteroids and unfractionated heparin	No interaction found	No interaction found	No interaction found	Monitor closely	Monitor closely

Abbreviation: DDIs, drug-drug interactions; DRV/c, darunavir-cobicistat.

In total, 575 DDIs for 58 drug pairs (305 associated with at least 1 ADR) were reported. Such DDIs were identified as follows: 70 by Medscape, 68 by COVID-19 Drug Interactions, 64 by Drugs.com, 55 by WebMD, and 48 by LexiComp. In 271 of 580 cases, no interactions were found. LexiComp reported the fewest DDIs, classified into B (no action needed) (10 [20%]), C (monitor therapy) (22 [45%]), D (consider modifying therapy) (10 [23%]), and X (avoid combinations) (6 [12%]).

The number of the identified severe-moderate DDI-associated adverse events was comparable among Drugs.com, Medscape, and WebMD. An equivalent classification was found using the latter 2 tools. Most DDIs were classified as major (30 [48%]) and moderate (32 [49%]) by Drugs.com, as serious (32 [46%]) and monitor closely (31 [44%]) by Medscape, and as serious (23 [43%]) and monitor closely (26 [46%]) by WebMD (Table 2). In addition, DDI-associated adverse events were classified as minor by Drugs.com in 2 cases (3%), by Medscape in 5 cases (7%), and by WebMD in 4 cases (7%).

COVID-19 Drug Interactions identified DDI-associated adverse outcomes as follows: 15 (22%) as do not coadminister, 32 (46%) as potential interaction, and 3 (4%) as potential weak interaction (Table 2). According to Medscape and WebMD, the most severe DDIs were caused by the association of amiodarone with lopinavir and ritonavir and lopinavir and ritonavir with simvastatin, classified as contraindicated (2 [3%]) by Medscape and as don’t use together (2 [4%]) by WebMD.

Globally, the reviewed studies described 15 patients taking lopinavir and ritonavir plus simvastatin^25^ and only 1 taking lopinavir and ritonavir plus amiodarone.^24^ The studies^24,25^ reported liver toxicity (related to lopinavir and ritonavir plus simvastatin) and orthostatic syncope (related to lopinavir and ritonavir plus amiodarone). For all the tools, besides these serious DDI-associated adverse outcomes already described, the remaining 301 can be divided into 117 (39%) classified as severe, 132 (43%) as moderate, and 52 (17%) as minor.

eTable 2 in the Supplement details the last step of the study. Of the 6917 studies identified, 20 studies, which enrolled 1297 patients, reported 115 DDI-related adverse events: 15 (26%) were identifiable by all tools analyzed, 29 (50%) were identifiable by at least 1 of them, and 14 (24%) remained nonidentifiable. Most of these involved psychotic disorders or cutaneous reactions.

## Discussion

Therapeutic strategy to treat COVID-19 has rapidly changed during the pandemic, above all based on experimental and real-world data and following the concept of repurposing. Some drugs have fallen out of use, whereas others represent a cornerstone of treatment.^2,36,37,38,39^ Both real-world data and results of clinical trials have highlighted the need to review all steps of the care process from the beginning of the pandemic to today.^37^ In particular, what seems clear is the large variability in the therapeutic response of patients with COVID-19 and therefore the urgent need to use a personalized approach.^38,39,40^ One important issue is that patients with comorbidities (thus polytreated), who represent most patients with COVID-19, are likely to experience ADRs, including those related to DDIs. Therefore, regardless of the drugs used for SARS-CoV-2 clearance and to treat COVID-19, it is crucial to take into account the risk of DDIs.^41^

The current study was planned to analyze DDI-associated clinical outcomes that occurred in clinical practice during the pandemic and to investigate whether and how drug interaction checkers might be useful to assess them. Our main finding is that the use of these tools could have identified several DDI-associated ADRs, including severe and life-threatening events. However, the interactions between the drugs used to treat COVID-19 and between the COVID-19 drugs and those already used by the patients should be evaluated.

At the beginning of the pandemic, chloroquine and hydroxychloroquine were largely used because of their ability (assessed in vitro) to modify cellular pH, thus interfering with SARS-CoV-2 replication and its fusion with the host cells.^2^ Then, as shown in the current study, hydroxychloroquine was recognized to interfere with the antiviral agents lopinavir-ritonavir, darunavir-cobicistat, and acetazolamide, causing QT-interval prolongation, ventricular arrhythmias, and torsade de pointes.^19,31^ In the study by Borba et al,^18^ several patients treated with chloroquine died after drug administration. Most patients (89.6%) with increased QT-interval prolongation were taking oseltamivir as well as acetazolamide and ceftriaxone. Crescioli et al^19^ reported 5 deaths among 23 patients. These patients had developed QT-interval prolongation after the coadministration of hydroxychloroquine with at least 1 of the following drugs: darunavir-cobicistat, acetazolamide, amiodarone, lopinavir-ritonavir, haloperidol, citalopram, and trazodone. Martínez-López-de-Castro et al^25^ reported that 3 of 44 deceased patients also had alteration of the QT interval associated with DDIs.

Lopinavir-ritonavir and darunavir-cobicistat were involved in most of the DDI-associated ADRs. Of importance, all the drug interaction checkers used in our study could have identified such events. This finding is not surprising, because these antivirals are inhibitors of cytochrome CYP3A4, which is the most involved isoenzyme of drug metabolism.

The interaction among hydroxychloroquine, darunavir-cobicistat, and tocilizumab can also lead to psychiatric disorders, such as behavioral disturbances, psychosis, agitation, delirium, and aggression. However, psychiatric ADRs were difficult to identify by the DDI tools. Martínez-López-de-Castro et al^25^ evaluated 2 patients taking hydroxychloroquine and lopinavir-ritonavir who experienced psychiatric disorders, whereas Anmella et al^16^ described 1 patient treated with acetazolamide, hydroxychloroquine, lopinavir-ritonavir, paroxetine, risperidone, and topiramate who had disturbing behavior.

None of the interaction tools identified the cutaneous ADRs that emerged from the systematic review. Martínez-López-de-Castro et al^25^ identified 8 patients with COVID-19 who reported cutaneous reactions following administration of acetazolamide plus hydroxychloroquine and hydroxychloroquine plus lopinavir-ritonavir. Skroza et al^30^ described erythematous rash, urticaria, and varicella-like blisters in 18 patients and 1 patient with a history of COVID-19 and late-onset urticarial vasculitis after healing.

Therapy must be chosen wisely, especially when dealing with drugs known to favor DDIs, such as anticoagulants.^42^ In this regard, Ghani et al^22^ described 3 patients treated with hydroxychloroquine and unfractionated or fractionated heparins or apixaban who had subarachnoid, severe cerebral edema, and intraparenchymal hemorrhages. A recent review^43^ also highlighted the risk of QT-interval prolongation and cardiomyopathy attributable to the possibility of interaction between apixaban and hydroxychloroquine because of a mechanism of inhibition of CYP2C8 and P-glycoprotein.

Several potential DDIs that involved anticancer drugs used for the treatment of COVID-19 were also found (eTable 1 in the Supplement). This finding is important considering that anticancer agents have a narrow therapeutic index and the ADRs are responsible for approximately 12% of hospitalizations in oncology units, almost 3 times more than in other medical areas.^44,45^ Anticancer drugs belonging to the targeted therapy are mainly associated with QT liability and interact with concomitant medications, increasing the likelihood of life-threatening ventricular arrhythmia.^43,46^ Nevertheless, our systematic review retrieved only 2 studies that reported potential DDIs that involved anticancer drugs. Szekely et al^31^ indicated a potential DDI that involved letrozole coadministered with chloroquine and memantine, leading to torsade de pointes. However, none of the 5 drug interaction checkers detected such a DDI. Treon et al^34^ documented a tachyarrhythmia potentially associated with acetazolamide, hydroxychloroquine, and ibrutinib administration. However, 4 of 5 drug interaction checkers recognized acetazolamide and hydroxychloroquine but not ibrutinib as responsible drugs for this DDI. No other DDI-associated adverse outcomes that involved ibrutinib were found despite this drug being a P-glycoprotein inhibitor and CYP3A4 substrate.^47^

The experience of the pandemic offers the opportunity to improve therapy for patients with other diseases, such as rheumatological diseases, who have variable responses to the disease-modifying antirheumatic drugs. Identifying pretherapeutic and on-treatment factors associated with drug effectiveness is essential in this field.^48^ The same goes for all drugs, including antivirals, anticoagulants, hypoglycemic agents, and antibiotics, whose use is not avoidable, especially in hospitalized patients. Recently, 2 oral antivirals were approved. One of them is molnupiravir, originally developed against influenza viruses.^49,50^ The other one is an association of 2 protease inhibitors, nirmatrelvir and ritonavir.^51^

Drug interaction checkers identified potential DDIs that involved nirmatrelvir-ritonavir and several drugs, such as colchicine, statins, antithrombotic, immunosuppressant, and antineoplastic agents, and DDIs that involved fluvoxamine combined with antidepressants, antiplatelet agents, benzodiazepines, and fentanyl. Conversely, only LexiComp identified a DDI between molnupiravir and cladribine. The reviewed studies^16,17,18,19,20,21,22,23,24,25,26,27,28,29,30,31,32,33,34,35^ did not report DDI-associated clinical outcomes, conceivably because of the recent use of these COVID-19 drugs. However, potential DDIs should never be underestimated. In particular, even if nirmatrelvir-ritonavir has been specifically developed for the treatment of COVID-19, the presence of ritonavir should be emphasized.

### Limitations

This study has some limitations. Only 5 (although widely used and consolidated) available drug interaction checkers were accessed, with the risk of overlooking some DDI-associated ADRs that occurred in clinical practice. However, the concomitant use of tools with different classification methods can complicate the assessment of the DDI-associated outcomes. Similarly, we may have neglected studies included in gray literature (eg, congress proceedings) and emerging sources (eg, preprint websites). Moreover, except for the study by Crescioli et al,^19^ which used the Naranjo algorithm, the other reviewed studies did not implement a causality assessment to ascertain the relationship between DDIs and the ADRs described. However, the aim of drug interaction checkers is to highlight the risk of DDI-associated ADRs to help physicians and patients to follow the most appropriate therapy and set up monitoring actions.

## Conclusions

The findings of this systematic review of drug interactions among patients with COVID-19 reported in databases and the literature suggest that extreme caution should be used in choosing COVID-19 therapy, especially in polytreated patients. Although a critical emergency, such as the COVID-19 pandemic, might justify an urgent clinical approach, possible DDIs should never be ignored when choosing the most effective and safest therapy. In this context, support could and can still derive from drug interaction checkers, which help to perform a therapeutic reconciliation by stopping use of or withholding drugs and by intensifying clinical monitoring. Attention must be paid to concomitantly examine different sources of information to manage old and new drugs. The COVID-19 pandemic offers learning and opportunity to draw on new ideas and stimuli to optimize the care of all patients with complex conditions.

## Notes

Supplement. **eFigure.** Flowchart of the Systematic Review **eTable 1. **Potential DDIs for Each Drug Administered Against COVID-19 With the Degree of Severity of the Associated Adverse Outcomes and/or ADRs Found by Using the Drug Interaction Checkers **eTable 2.** Reviewed Studies With Reported DDIs and Their Predictability (YES or NO) by Using the DDI Checkers
