# Supplementary material for: Identification of Drug Interaction Adverse Events in Patients With COVID-19: A Systematic Review
Source: JAMA Netw Open. 2022 Apr 19;5(4):e227970. doi: 10.1001/jamanetworkopen.2022.7970 (PMC9020212; doi:10.1001/jamanetworkopen.2022.7970)
Supplement: Supplement. — eFigure. Flowchart of the Systematic Review eTable 1. Potential DDIs for Each Drug Administered Against COVID-19 With the Degree of Severity of the Associated Adverse Outcomes and/or ADRs Found by Using the Drug Interaction Checkers eTable 2. Reviewed Studies With Reported DDIs and Their Predictability (YES or NO) by Using the DDI Checkers [file jamanetwopen-e227970-s001.pdf]

Supplementary Online Content

Conti V, Sellitto C, Torsiello M, et al. Identification of drug interaction adverse events in patients with COVID-19: a systematic review. *JAMA Netw Open*. 2022;5(4):e227970. doi:10.1001/jamanetworkopen.2022.7970

eFigure. Flowchart of the Systematic Review

eTable 1. Potential DDIs for Each Drug Administered Against COVID-19 With the Degree of Severity of the Associated Adverse Outcomes and/or ADRs Found by Using the Drug Interaction Checkers

eTable 2. Reviewed Studies With Reported DDIs and Their Predictability (YES or NO) by Using the DDI Checkers

This supplementary material has been provided by the authors to give readers additional information about their work.

© 2022 Conti V et al. *JAMA Network Open.*

eFigure. Flowchart of the Systematic Review

PRISMA 2009 Flow Diagram

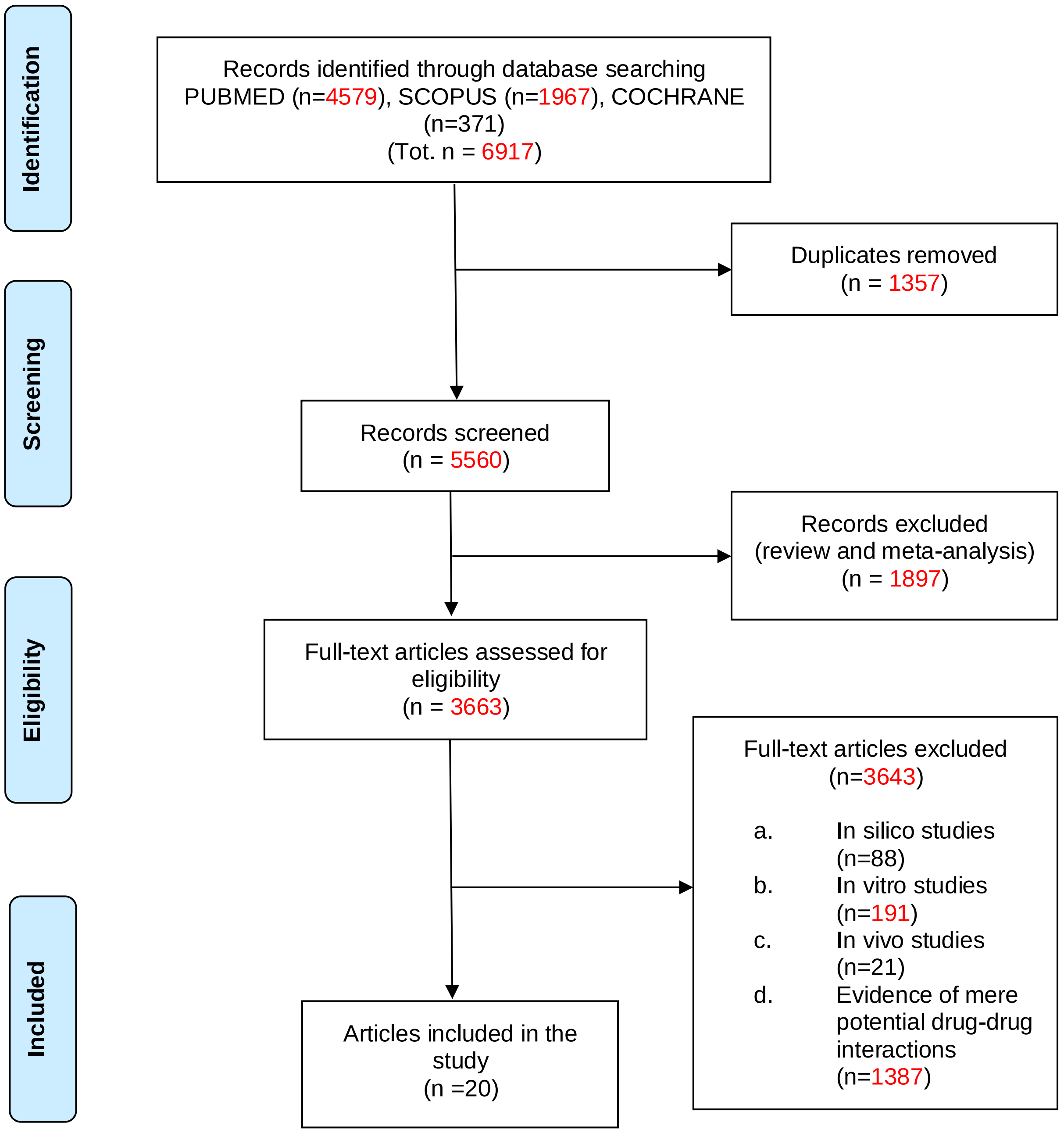

© 2022 Conti V et al. *JAMA Network Open.*

eTable 1. Potential DDIs for Each Drug Administered Against COVID-19 With the Degree of Severity of the Associated Adverse Outcomes and/or ADRs Found by Using the Drug Interaction Checkers

Drug	No. of DDIs by drug interaction checkers																	Total No. of DDIs
	Drugs.com			Liverpool COVID-19 DDI			LexiComp				Medscape				WebMD			
	Major	Moderate	Minor	Do not co-administer	Potential interaction	Potential weak interaction	Avoid combination	Consider therapy modification	Monitor therapy	No action needed	Contra-indicated	Serious; use alternative	Monitor closely	Minor	Severe	Serious	Moderate	
Acalabrutinib	189	203	0	NA	NA	NA	20	15	30	0	0	63	168	0	3	18	4	713
Anakinra	33	285	7	8	9	29	10	9	9	0	2	63	21	1	5	7	1	499
Azithromycin	64	224	21	24	94	12	10	13	47	11	1	38	140	50	16	51	40	856
Baricitinib	229	58	6	8	12	3	11	12	16	0	0	52	4	0	1	3	0	415
Canakinumab	32	285	7	9	8	28	10	9	9	0	0	60	18	0	3	6	1	485
Casirivimab-imdevimab	0	6	0	NA	1	NA	NA	NA	NA	NA	0	NA	NA	NA	NA	NA	NA	7
Chloroquine	67	296	15	35	142	17	11	21	68	16	10	38	126	97	16	60	23	1058
Cobicistat	166	216	16	NA	NA	NA	98	125	109	31	22	72	120	1	38	107	36	1157
Cobicistat-darunavir	178	323	17	NA	NA	NA	122	157	159	39	NA	NA	NA	NA	43	118	37	1193
Colchicine	85	116	5	NA	NA	NA	6	8	25	5	0	73	65	2	0	5	5	400
Darunavir	80	312	18	NA	NA	NA	93	136	138	37	28	102	729	41	27	43	30	1814
Defibrotide	61	67	7	NA	NA	NA	1	6	36	0	16	0	42	0	2	5	3	246
Dexamethasone	117	507	51	1	97	3	24	36	63	0	19	123	312	124	6	27	17	1527
Emapalumab	17	58	0	NA	NA	NA	7	9	10	0	0	0	4	0	3	5	1	114
Enoxaparin	92	82	7	NA	NA	NA	9	6	39	0	3	63	139	17	3	11	12	483
Bamlanivimab-etesevimab	NA	NA	NA	NA	1	NA	0	3	0	0	0	NA	NA	NA	NA	NA	NA	4
Favipiravir	NA	NA	NA	0	14	37	0	1	2	7	NA	NA	NA	NA	NA	NA	NA	61
Fluvoxamine	112	537	19	26	228	22	20	23	133	20	6	114	301	0	14	28	47	1650
Hydroxychloroquine	60	268	5	35	142	17	3	2	20	6	1	171	17	2	4	7	3	763
Ibuprofen	92	255	14	NA	NA	NA	9	24	67	0	0	28	247	101	4	13	26	880
Interferon beta 1A	12	287	1	NA	NA	NA	1	0	1	0	0	3	9	1	3	15	2	335
Interferon beta 1B	12	287	1	1	14	1	1	0	1	0	0	3	7	0	3	15	2	348
Ivermectin	0	75	2	0	45	4	2	2	3	0	0	5	49	0	NA	NA	NA	187
Ketoprofen	91	241	9	NA	NA	NA	9	23	66	0	0	19	236	73	4	13	23	807
Lopinavir-ritonavir	223	431	36	52	208	49	123	173	162	53	37	156	438	34	45	135	59	2414

© 2022 Conti V et al. *JAMA Network Open.*

Mavrilimumab	NA	NA	NA	NA	NA	NA	NA	NA	NA	NA	NA	NA	NA	NA	NA	NA	NA	0
Methylprednisolone	79	364	37	NA	NA	NA	16	27	58	13	1	70	242	119	3	15	14	1058
Molnupiravir	NA	NA	NA	NA	NA	NA	1	0	0	0	NA	NA	NA	NA	NA	NA	NA	1
Nirmatrelvir-ritonavir	220	355	38	53	131	50	124	160	230	79	32	16	57	0	43	131	52	1771
Opaganib	NA	NA	NA	NA	NA	NA	NA	NA	NA	NA	NA	NA	NA	NA	NA	NA	NA	0
Pamrevlumab	NA	NA	NA	NA	NA	NA	NA	NA	NA	NA	NA	NA	NA	NA	NA	NA	NA	0
Paracetamol	18	74	35	NA	NA	NA	0	4	20	0	0	3	24	50	0	1	3	232
Regdanvimab	NA	NA	NA	NA	NA	NA	NA	NA	NA	NA	NA	NA	NA	NA	NA	NA	NA	0
Remdesivir	NA	NA	NA	9	0	0	NA	NA	NA	NA	NA	NA	NA	NA	0	1	0	10
Reparixin	NA	NA	NA	NA	NA	NA	NA	NA	NA	NA	NA	NA	NA	NA	NA	NA	NA	0
Ritonavir	187	343	41	NA	NA	NA	100	147	149	44	27	123	356	70	36	71	37	1731
Ruxolitinib	84	224	43	8	66	8	13	15	27	5	0	25	42	102	3	6	1	672
Sarilumab	40	134	7	8	9	29	8	9	10	0	0	13	21	0	4	8	3	303
Selinexor	33	78	0	NA	NA	NA	10	10	13	1	0	163	0	0	3	7	1	319
Siltuximab	34	136	7	NA	NA	NA	11	10	16	0	0	2	31	0	1	3	0	251
Solnatide	NA	NA	NA	NA	NA	NA	NA	NA	NA	NA	NA	NA	NA	NA	NA	NA	NA	0
Sotrovimab	0	6	0	NA	NA	NA	0	3	0	0	NA	NA	NA	NA	0	0	1	10
Tocilizumab	40	206	7	8	9	29	9	9	10	0	0	62	24	2	4	8	1	428

Abbreviations: ADRs, adverse drug reactions; DDI, drug-drug interaction; NA, not available.

© 2022 Conti V et al. *JAMA Network Open.*

eTable 2. Reviewed Studies with reported DDIs and their predictability (YES or NO) by using the DDI-checkers. Last accessed February 13th, 2022.

Reviewed studies	Drugs involved in potential DDIs	DDIs-related adverse clinical outcomes and/or ADRs	Drugs.com	COVID-19 Drug Interactions	Lexicomp	Medscape	Webmd
Anmella G. et al. (2020)[18]	AZM + HCQ + LPV/r + paroxetine + risperidone + topiramate	Behavioral disturbances	**NO**	**NO**	**YES**	**YES**	**YES**
Anmella G. et al. (2020)[18]	AZM + HCQ + LPV/r + venlafaxine	Mild QT interval prolongation (443 ms)	**YES**	**YES**	**YES**	**YES**	**YES**
Bartiromo M. et al. (2020)[19]	DRV/c + HCQ + tacrolimus	Tacrolimus trough levels turned to be extremely high (90.5 ng/mL), intermittent abdominal pain, nausea and vomit	**YES**	**YES**	**YES**	**YES**	**NO**
Borba M. G. S. et al. (2020)[20]	AZM + ceftriaxone + CQ	Rhabdomyolysis, myocarditis, severe arrhythmias, QT interval prolongation	**YES**	**YES**	**YES**	**YES**	**YES**
Borba M. G. S. (2020)[20] et al.	AZM + ceftriaxone + CQ + oseltamivir	Rhabdomyolysis, myocarditis, severe arrhythmias, QT interval prolongation	**YES**	**YES**	**YES**	**YES**	**YES**
Crescioli, G. et al. (2020)[21]	Amiodarone + AZM + DRV/c + HCQ	QT interval prolongation	**YES**	**YES**	**YES**	**YES**	**YES**
Crescioli, G. et al. (2020)[21]	AZM + citalopram + HCQ + LPV/r	QT interval prolongation	**YES**	**YES**	**YES**	**YES**	**YES**
Crescioli, G. et al. (2020)[21]	AZM + DRV/c + HCQ	Diarrhoea, vomiting	**YES**	**NO**	**NO**	**NO**	**NO**
Crescioli, G. et al. (2020)[21]	AZM + haloperidol + HCQ + levomepromazine + LPV/r + zuclopenthixol	QT interval prolongation	**YES**	**YES**	**YES**	**YES**	**YES**
Crescioli, G. et al. (2020)[21]	AZM + haloperidol + HCQ + LPV/r	QT interval prolongation	**YES**	**YES**	**YES**	**YES**	**YES**
Crescioli, G. et al. (2020)[21]	AZM + HCQ + LPV/r	QT interval prolongation	**YES**	**YES**	**YES**	**YES**	**YES**
Crescioli, G. et al. (2020)[21]	AZM + HCQ + LPV/r	QT interval prolongation, vomiting	**YES**	**YES**	**NO**	**YES**	**YES**
Crescioli, G. et al. (2020)[21]	AZM + HCQ + sertraline	QT interval prolongation	**YES**	**YES**	**YES**	**YES**	**YES**
Crescioli, G. et al. (2020)[21]	Citalopram + HCQ	QT interval prolongation	**YES**	**YES**	**YES**	**YES**	**YES**
Crescioli, G. et al. (2020)[21]	DRV/c + haloperidol + HCQ	QT interval prolongation	**YES**	**YES**	**YES**	**YES**	**YES**
Crescioli, G. et al. (2020)[21]	DRV/c + HCQ	QT interval prolongation	**NO**	**NO**	**NO**	**NO**	**NO**
Crescioli, G. et al. (2020)[21]	DRV/c + HCQ + LPV/r + tocilizumab	Hypertransaminasemia	**NO**	**NO**	**NO**	**NO**	**NO**
Crescioli, G. et al. (2020)[21]	DRV/c + HCQ + tocilizumab	Psychosis, agitation, delirium, aggressiveness	**NO**	**NO**	**YES**	**NO**	**NO**
Crescioli, G. et al. (2020)[21]	DRV/c + tacrolimus	Nausea, vomiting, abdominal pain, drug level modification	**YES**	**NO**	**YES**	**NO**	**NO**
Crescioli, G. et al. (2020)[21]	HCQ + LPV/r	QT interval prolongation, hypokalaemia	**YES**	**YES**	**NO**	**YES**	**NO**

© 2022 Conti V et al. *JAMA Network Open.*

Crescioli, G. et al. (2020)[21]	HCQ + LPV/r	QT interval prolongation	**YES**	**YES**	**NO**	**YES**	**NO**
Crescioli, G. et al. (2020)[21]	HCQ + magnesium sulphate	QT interval prolongation, hypokalaemia	**NO**	**NO**	**NO**	**NO**	**NO**
Crescioli, G. et al. (2020)[21]	HCQ + risperidone	QT interval prolongation, atrial flutter, hemiplegia, hypokalaemia, major depression	**YES**	**YES**	**YES**	**YES**	**YES**
Crescioli, G. et al. (2020)[21]	HCQ + trazodone	QT interval prolongation	**YES**	**YES**	**NO**	**NO**	**NO**
Elton Dajti et al. (2020)[22]	DRV/c + HCQ + prednisone + tacrolimus	Increased tacrolimus levels	**YES**	**YES**	**YES**	**YES**	**YES**
Gautret P. et al. (2021)[23]	AZM + HCQ	QT interval prolongation (>60ms), discontinuation of treatment	**YES**	**YES**	**NO**	**YES**	**YES**
Ghani MU. et al. (2020)[24]	Apixaban + enoxaparin + HCQ + steroids	A large intraparenchymal hemorrhage and cerebral edema	**YES**	**YES**	**YES**	**NO**	**NO**
Ghani MU. et al. (2020)[24]	Apixaban + HCQ + steroids + unfractionated heparin	Scattered subarachnoid hemorrhages, a subdural hematoma	**YES**	**NO**	**YES**	**NO**	**NO**
Ghani MU. et al. (2020)[24]	HCQ + steroids + unfractionated heparin	Acute subarachnoid and intraparenchymal hemorrhages within the posterior fossa	**NO**	**NO**	**NO**	**NO**	**NO**
Li X. et al. (2020)[25]	Ganciclovir + LPV/r + oseltamivir + peramivir + penciclovir + rubavirin + umifenovir	Increase in D-dimer, haematological abnormalities	**NO**	**NO**	**NO**	**NO**	**NO**
Macías, J. et al.(2020)[26]	Amiodarone + LPV/r	Orthostatic syncope	**YES**	**NO**	**YES**	**NO**	**NO**
Martínez-López-de-Castro N. et al. (2020)[27]	Alprazolam + LPV/r	Psychiatric Disorders	**NO**	**NO**	**YES**	**NO**	**NO**
Martínez-López-de-Castro N. et al. (2020)[27]	Aripiprazole + digoxin + fentanyl + lithium + LPV/r + tacrolimus	Alteration of the concentration of blood levels	**YES**	**YES**	**YES**	**YES**	**YES**
Martínez-López-de-Castro N. et al. (2020)[27]	AZM + HCQ	Cutaneous reactions	**NO**	**NO**	**NO**	**NO**	**NO**
Martínez-López-de-Castro N. et al. (2020)[27]	AZM + LPV/r	Gastrointestinal Disorders	**YES**	**NO**	**NO**	**NO**	**NO**
Martínez-López-de-Castro N. et al. (2020)[27]	HCQ + LPV/r	Hyperglicaemia	**NO**	**NO**	**NO**	**NO**	**NO**
Martínez-López-de-Castro N. et al. (2020)[27]	HCQ + LPV/r	Cutaneous reactions	**NO**	**NO**	**NO**	**NO**	**NO**

© 2022 Conti V et al. *JAMA Network Open.*

Martínez-López-de-Castro N. et al. (2020)[27]	HCQ + LPV/r	Gastrointestinal disorders	**YES**	**NO**	**NO**	**NO**	**NO**
Martínez-López-de-Castro N. et al. (2020)[27]	HCQ + LPV/r	Psychiatric Disorders	**NO**	**NO**	**NO**	**NO**	**NO**
Martínez-López-de-Castro N. et al. (2020)[27]	HCQ + tacrolimus	Alteration of the concentration of blood levels	**NO**	**NO**	**NO**	**NO**	**NO**
Martínez-López-de-Castro N. et al. (2020)[27]	Interferon beta + metamizole	Haematological Toxicity	**NO**	**YES**	**NO**	**NO**	**NO**
Martínez-López-de-Castro N. et al. (2020)[27]	LPV/r + methylprednisolone or prednisone	Hyperglicaemia	**NO**	**NO**	**YES**	**NO**	**NO**
Martínez-López-de-Castro N. et al. (2020)[27]	LPV/r + midazolam or diazepam	Increased sedative effect	**YES**	**YES**	**YES**	**YES**	**NO**
Martínez-López-de-Castro N. et al. (2020)[27]	LPV/r + propofol	Increased triglyceride level	**NO**	**NO**	**NO**	**NO**	**NO**
Martínez-López-de-Castro N. et al. (2020)[27]	LPV/r + simvastatina	Liver toxicity	**NO**	**NO**	**NO**	**YES**	**NO**
Martínez-López-de-Castro N. et al. (2020)[27]	LPV/r + valproate	Seizures	**NO**	**NO**	**YES**	**NO**	**NO**
Meriglier, E. et al. (2021)[28]	Darunavir/ritonavir + HCQ	Diarrhoea grade I and II; ECG abnormalities; hepatic enzyme elevation	**NO**	**NO**	**NO**	**YES**	**YES**
Meriglier, E.et al. (2021)[28]	HCQ + LPV/r	Diarrhoea grade I and II; ECG abnormalities; severe nausea	**YES**	**YES**	**NO**	**YES**	**NO**
Meziyerh S. et al. (2020)[29]	Everolimus + HCQ + LPV/r	Dyspnoea/tachypnea, everolimus plasma concentrations increased.	**YES**	**YES**	**YES**	**YES**	**NO**
Nham E. et al. (2020)[30]	Ceftriaxone + levofloxacin + LPV/r	Severe thrombocytopenia with epistaxis and petechiae.	**NO**	**NO**	**NO**	**NO**	**NO**
Ramireddy A. et al. (2020)[31]	AZM + HCQ	QT interval prolongation	**YES**	**YES**	**YES**	**YES**	**YES**
Skroza N. et al. (2020)[32]	Ceftriaxone + enoxoparin + HCQ + LPV/r	Urticarial vasculitis due to adverse drug reaction	**NO**	**NO**	**NO**	**NO**	**NO**
Szekely Y. et al. (2020)[33]	CQ + letrozole + memantine	Extreme QT interval prolongation (720 ms), torsades de pointes	**NO**	**NO**	**NO**	**NO**	**NO**

© 2022 Conti V et al. *JAMA Network Open.*

Teoli D. et al.(2021)[34]	Remdesivr + tramadol	Severe pain localized in the legs	**NO**	**NO**	**YES**	**NO**	**NO**
Thammathiwat T. et al (2021)[35]	Darunavir/ritonavir + tacrolimus	Tacrolimus levels turned significantly high, acute kidney injury, lymphopenia, PiO2/FiO2 was lowered, tacrolimus withdrawn for 10 days	**YES**	**NO**	**YES**	**YES**	**YES**
Treon SP. et al. (2020)[36]	AZM + HCQ + ibrutinib	Wide QRS complex tachyarrhythmia	**YES**	**YES**	**NO**	**YES**	**YES**
Yekedüz E. et al. (2020)[37]	Anti-diabetics + HCQ	Hypoglycemia	**NO**	**NO**	**YES**	**NO**	**NO**

© 2022 Conti V et al. *JAMA Network Open.*
